# Patients’ experiences of lifestyle discussions based on motivational interviewing: a qualitative study

**DOI:** 10.1186/1472-6955-13-13

**Published:** 2014-05-05

**Authors:** Elisabeth Brobeck, Sigrid Odencrants, Håkan Bergh, Cathrine Hildingh

**Affiliations:** 1 Department of Research, Development and Education, Halmstad, Sweden; 2 School of Health and Medical Sciences Örebro University, Örebro, Sweden; 3 Department of Research, Development and Education, Varberg, Sweden; 4 School of Social and Health Sciences, Halmstad University, Halmstad, Sweden

**Keywords:** Content analysis, Lifestyle discussion, Motivational interviewing, Nurse, Patient experiences, Primary health care

## Abstract

**Background:**

According to World Health Organization about 75% of cardiovascular diseases and type 2 diabetes and 40% of all cases of cancer could be prevented if the risk factors tobacco use, unhealthy diets, physical inactivity and harmful use of alcohol could be eliminated. Patients often need help in monitoring themselves to make the proper lifestyle changes and it is important that adequate support is provided to enable the patients to take control over their health. Motivational interviewing is a framework that can help to facilitate this movement. The aim of this study was to describe how patients in primary health care settings experience lifestyle discussions based on motivational interviewing.

**Methods:**

This study has a descriptive design and qualitative content analysis was used as the method. Sixteen patients who had each visited a registered nurse for lifestyle discussions were interviewed.

**Results:**

The results show that the lifestyle discussions could enable self-determination in the process of lifestyle change but that certain conditions were required. Mutual interaction between the patient and the nurse that contributes to a sense of well-being in the patients was a necessary condition for the lifestyle discussion to be helpful. When the discussion resulted in a new way of thinking about lifestyle and when patient initiative was encouraged, the discussion could contribute to change. The patient’s free will to make a lifestyle change and the nurse’s sensitivity in the discussions created fertile soil for change.

**Conclusions:**

This study focuses on MI-based discussions, and the result shows that a subset of patients, who self-reported that they are motivated and aware of their role in making lifestyle changes, appreciate these strategies. However, it is not known whether discussions would be experienced in the same way if RNs used another method or if patients who were less motivated, engaged, or aware of their role in making lifestyle changes were interviewed.

## Background

Non-communicable diseases (NCDs) such as cardiovascular diseases, type 2 diabetes, cancers and chronic respiratory diseases are by far the leading cause of death globally, representing 63% of all annual deaths [1]. NCDs are largely caused of the four risk factors; tobacco use, unhealthy diets, physical inactivity and harmful use of alcohol [1,2]. According to WHO (World Health Organization) about 75% of cardiovascular diseases and type 2 diabetes and 40% of all cases of cancer could be prevented if these risk factors could be eliminated [1]. Therefore it is important that patients obtain support and is given an opportunity to control their lifestyle and take responsibility for their health [3,4]. Lifestyle changes are often more difficult than expected and are also difficult to maintain [5,6]. If patients want to change their lifestyles they must gain knowledge and understanding of their problems [6,7] To truly support people undergoing lifestyle changes meetings with the patients must involve dialogue about how they feel, think and act regarding their lifestyle [6,8,9]. Registered Nurses (RNs) are one category of health professionals who can be important agents in work with lifestyle issues [10-12].

Motivational Interviewing (MI) is a patient-centered method that aims to promote healthy behaviour changes and a framework that can help facilitate lifestyle changes [13,14]. MI demands that the RNs interact with the patients and listen to their opinions about why they want the change, why a change might be good and how they can succeed. Patients often have conflicting feelings about a change when they both want and do not want to realize the change. Central to MI is helping people resolve this ambivalence, by focusing on a patient’s own perception and motivation. When MI works, the RN speaks in a way that minimizes resistance and encourages discussions about the change and the patient’s commitment, increasing the chances that he or she will actually achieve the change [13,14].

MI consists of five specific techniques: open-ended questions, reflective listening, affirmations, summarizing and eliciting. However over the time, the emphasis has been increasingly on the spirit of MI based on collaboration, evoking and autonomy [13,15].

RNs, like other health care professionals, have a responsibility in promoting health, and in Sweden RNs are heavily involved in health promotion practice (HPP) [16]. It is essential that they understand how they can effectively help people who have lifestyle problems [6,7,17]. Research has shown that RNs experience MI as a valuable tool in their HPP [18] and several studies indicate that MI is an effective method to support patients to make lifestyle changes [19-23]. However, meta-analysis of MI has reached different conclusions, due to insufficiently considered fidelity of the treatment that was provided in the study. Miller & Rollnick [24] point out the importance to clarify the conditions under which a complex treatment like MI is less or more effective.

Studies are lacking on how patients experience lifestyle discussions in which nurses utilize MI. It is therefore important that patients’ experiences of lifestyle discussions be considered to increase knowledge of the aspects that are relevant to the choice to change lifestyle. This can make nurses more aware of what is important when they use MI to support patients’ attempts to make lifestyle changes.

### Aim

The aim of the study was to describe how patients in primary health care settings experience lifestyle discussions based on MI.

## Methods

This study, which has a descriptive design and uses a qualitative method, was conducted in primary health care in a county in southwestern Sweden.

### Sample

Twenty RNs who had been interviewed earlier about their experiences with MI as a method for HPP [18] mediated names of patients who had visited them for lifestyle discussions. The RNs worked in 13 different health centres that included district nurse surgery centres and doctor surgery centres all over the county in either urban or rural areas and with different population structure. The RNs, all female, had 12-40 years of professional experience. The whole group of RNs worked continuously with lifestyle discussions among their patients. All of the RNs had taken part in training sessions of MI, and they had been practicing MI for 3-10 years. At the time, nine of the RNs were seeing patients for lifestyle discussions. The RNs was instructed that during one month ask the two first patients they had met at least three times for such discussions whether they wanted to participate in the study. Two RNs were able to contact one patient each, and the other seven contacted two patients each. All of these patients agreed to participate in the study, altogether, sixteen patients (six women and ten men), ranging from 28 to 77 years old with an average of 60 years. Seven patients had histories of smoking, one with both smoking and weight-control problems, four had histories of weight-control problems and four were at risk for alcohol abuse. The patients had visited their respective RNs for lifestyle discussions at least three times and up to several times during four years. All patients were Swedish speaking.

### Data collection

Data were collected using an open, qualitative interview [25] in the form of a dialogue. The interview began with a question about what in their lifestyle the patient wanted to change. Then they were asked to talk about their experiences of the lifestyle discussion with the RN on three areas; contact, progress and opportunity/barriers. The question was about the contact with the RN in the lifestyle discussions, the progress they made and the opportunities and barriers they experienced in the lifestyle discussions with the RN. The follow-up questions were based on what occurred during the interview. The same person in the research team conducted all the interviews. The interviews lasted from 30 to 45 minutes and were tape-recorded for later transcription by the interviewer.

### Data analysis

The data were processed using a qualitative content analysis that consisted of a number of analytical steps as described by Graneheim and Lundman [26]. The entire text of each interview was studied several times to obtain a holistic impression of the content. The sentences and phrases, or “meanings units”, that corresponded to the study’s purpose were selected. The meaning units were then condensed to shorten the text while maintaining the essential content. The condensed meaning units were then abstracted, coded, and structured on the basis of similar content into subcategories and categories. The categories reflected the central message in the interviews and constituted the apparent content. Finally, a theme that indicated an interpretation of the text emerged from the latent content [26].

### Ethical considerations

The regional ethical review board in Lund, Sweden (dnr 276/2008), approved the study, and the data were collected in accordance with the Helsinki Declaration [27]. The operation manager at the medical centre where the patient has been on lifestyle discussions accepted that the RN disclosed the patient’s name and telephone number to the interviewer. The participants, who received both oral and written information about the study and provided their written consent to participate. The participants were aware that their participation was voluntary and that they had the option to cancel the interview at any time with no obligation to provide a reason and without affect on their contact with the RN. The patient also received information that there would be no feedback of the interview to “their” RN. The participants were in no manner dependent on the interviewer. Participants were informed that the data would be treated confidentially. All details about the patients come from their own accounts.

## Results

The lifestyle discussions between patient and RN required certain conditions in order to be meaningful to the patient and to lead to possible lifestyle changes. First, mutual interaction was necessary in which the patients felt that the RN was present and listened to them in a genuine way. In addition, the patients themselves needed a true desire to implement a lifestyle change in order to succeed. Without this desire and without self-determination, there are no good prospects for change. The results also show that a successful lifestyle discussion between patient and RN created a sense of well-being; patients experienced support from the RNs and felt they were not alone with their problems. The patients felt satisfaction and motivation to manage a lifestyle change. They felt confirmed when they were met with respect instead of pointers. When the discussion resulted in a new way of thinking about lifestyle, and when patients’ own initiatives were encouraged, the discussion could contribute to change. The categories *presume mutual interaction*, *create a sense of well-being*, and *contribute to change* describe how patients in the primary-care settings experienced lifestyle discussions in which the RNs utilized MI. The overall theme is that the lifestyle discussions enabled self-determination. The categories and subcategories are presented below (Table 1).

**Table 1:** Patients’ view of lifestyle discussions

**Theme**	**Category**	**Subcategory**
Enabling self-determination	Presume mutual interaction	Presume nothing
		Presume commitment to patient
	Create a sense of well-being	Provide support
		Provide satisfaction
		Provide confirmation
		Provide no feelings of guilt
	Contribute to change	Encourage other thoughts
		Encourage own initiatives

### Presume mutual interaction

The patients were fully aware that they needed to be willing to change on their own and that the RN would not press them to succeed. Instead, the RN they would encourage self-determination. The importance of creating a confidential relationship built on trust and confidence between patient and RN was highlighted. This mutual interaction was seen as necessary for facilitating a lifestyle change.

### Presume nothing

The patients expressed the importance of their own free will to change. They also stated that it was difficult to make lifestyle changes and that they knew they had to do the job themselves, taking responsibility for it. A patient said:

*I think you must come to an understanding about yourself……decide for yourself……and that getting help is another matter.* (Patient 16)

Patients were of the opinion that a RN needed to understand the patient’s own thoughts about the lifestyle change, know what worked best for her or him personally, and recognize that not everyone can or wants to make a change in the same way. If the RN demands were too high or if she tried to convince a patient to accept her opinions, the patient could easily lose the motivation to change.

### Presume commitment to patient

A great advantage for the patients was that the RN was present and took the time to listen carefully to their problems. Often feelings of shame are associated with lifestyle problems; therefore the RN had to be sensitive in order to allow a meaningful discussion. The patients needed to feel sure that their stories were important not only to themselves but also to the RN and that she was truly engaged in their problems. A patient described this as:

*Firstly, the nurse has to have an instinctive feeling because we are all different. She must be able to feel…… be very perceptive, at least in the beginning so that she get to know what sort of mentality the person she is talking to has……as it is then easier to talk about certain things.* (Patient 16)

In a close relationship the patients felt that they could be honest and open. Talking to another person about their problems was a daunting step for many of the patients, but when they had the courage to talk, they felt liberated. A patient expressed:

*I felt I could be honest with her……if I felt bad I could say so, and if I felt fine I could say so. I could tell her about my experiences and what had happened, what I had done, and such things that I had been very ashamed of.* (Patient 9)

### Create a sense of well-being

When the lifestyle discussions were perceived as meaningful and open for self-determination, they provided support, satisfaction, and confirmation, which in turn created a feeling of well-being for the patients. This feeling was important for initiating a positive life-changing process. It was also important that patients did not experience any feelings of guilt about their behavior when they discussed their problems with the RN.

### Provide support

The patients did not feel that they were alone with their problems if they received strong support from the RN. They experienced a need to talk and felt alone in the endeavor to change without the discussions. Extremely important for the patients was not only what was said but also that they worked with the RN to reach their goals. A patient pointed out:

*It felt as though we did it together…even though the nurse did not do it…and that felt amazingly good.* (Patient 7)

Without support from the nurses, it would have been easy to delay the lifestyle change. The patients emphasized that it was easy to think that they could manage the change on their own but also that it was often more difficult than they had imagined, especially if they lacked support. A patient illustrated the difficulty as:

*It absolutely felt as if I could not deal with this myself. I won’t….. I must have someone to support me.* (Patient 2)

It was most beneficial to have help from outsiders in making the change. Close friends and family sometimes nagged or held unrealistically high expectations, and many became disappointed if the patient did not succeed. At times, the patients also felt ashamed of their behavior in front of close family members. According to a patient:

*It’s better to go to an outsider than to someone who is very much involved, because that is not the same thing.* (Patient 16)

The patients discovered that RNs who work with patients with a desire to make lifestyle changes have insight into what it means to make a change. The RNs know how hard it is, whereas this is not always the case with close family members.

### Provide satisfaction

The discussion made the patients feel happy and motivated, and it increased their self-determination in making the change. At the same time, they emphasized that it was not necessary to have a long and complicated discussion; the important thing was that the RN was there and cared about them. A patient said:

*When I got back home, I went through everything we had talked about, and it felt really good. I was really happy…the nurse radiates real positivity and made me feel that I could easily fix this.* (Patient 7)

### Provide confirmation

The patients felt that the RN accepted them despite their problems, that the RN understood their thoughts during the changing process. It was important to the patients to receive confirmation that they were on the right path in the changing process of changing. A patient described this as:

*The nurse understands me when I say what I think…… not just someone saying, “You cannot carry on with that”….. It doesn’t work. You must be understood; otherwise, it doesn’t help.* (Patient 13)

### Provide no feelings of guilt

The patients experienced that instead of imposing guilt, the discussions created a positive and nonjudgmental feeling. They pointed out that if they had felt offended or that they should blame themselves for their harmful lifestyle, they would have ceased attending the consultations. The patients did not experience moral preaching during the discussions, and they said that they would not have listened to scare tactics. They thought that they were met with respect even when the change did not succeed; that relapse was accepted also gave patients the courage to admit relapses. A patient mention this as:

*You get respect even if you don’t succeed, because it is normal to fail with things now and again.* (Patient 4)

### Contribute to change

The ability to think differently about how the change could be made and to have the self-determination to achieve the changes was important to the change process. If prerequisites indicated that the discussion would be worthwhile and if the patients experienced the discussions as positive the change process would be easier.

### Encourage other thoughts

The patients were often well aware of the right lifestyle choices, but the most important factor was being able to see their problem in another way. Therefore they needed help to change their thoughts when these had become stuck in a specific pattern. Without help, it was more difficult for them to emerge from this pattern and to make a change. According to a patient:

*If you want help and go to these talks, then you have taken away this obstacle a little, in any case. If you continue, perhaps the obstacle disappears altogether.* (Patient 2)

Talking about their problems with making a change and putting them into words caused the patients’ doubts to disappear.

### Encourage own initiatives

The patients had the opportunity to take initiative and make their own decisions about the change process. They were allowed to talk about what they wanted to do and how it would be done while the RN listened without lecturing. The patients did not find that the discussions were meant to correct their ideas or that any pressure was put on them. If they were criticized in a discussion, they would not participate in further discussions, even after weighing the pros and cons of a lifestyle change. It was easy to be oversensitive to criticism or pressure, and if patients perceived any, they stop listening. A patient pointed out:

*I was not being lectured like “Stop that”, “Do this or that”, but she listens more. I thought that it was very positive.* (Patient 3)

## Discussion

Central to MI is helping patients resolve their ambivalence by, for instance focusing on a patient’s own perception and motivation [13-15]. Of course wanting and not wanting to change lifestyle is conflicting. But the conflict may also be that the patients want to change, but lack the tools and motivation to do so. The results of the study show that patients often are well aware of the right lifestyle choices, but feelings of shame are associated with lifestyle problems. Talking to another person about their problems was a significant step for many of the patients, and they pointed out that if they had felt offended or that they themselves were to blame for the problem, they would have stopped attending the consultations. Previous studies have also shown that patients often have sufficient knowledge about the risks connected to their lifestyles, especially smoking and being overweight, and that they understand the consequences to their health [5,28].

Since the aim of this study was to describe how patients experienced the lifestyle discussions, the patients were asked about the discussion itself and not of the effects. During the interviews, the patients spoke about what they generally considered necessary for a meaningful discussion and what they had gained from it, providing a broad picture of their thoughts. Nevertheless, our results indicate that the patients highlighted several aspects in the spirit of MI that contribute to change. For example, mutual interaction was seen as necessary in facilitating a lifestyle change. In order to motivate the patients to carry out the changes, the RNs needed to talk with the patients and not at them. The patients appreciated coming to the sessions, during which they received motivation and praise for small and large steps forward. This finding was verified by Bowden et al*. *[29] who studied the process of changing health-risk behaviors and found that praise for progress was valuable in the process of change. Moyers et al*. *[30] also described how the advisers’ ability to cooperate with the patients was important in enabling them to open up about themselves and their problems. This was apparent in our study, as most participants indicated that the confidentiality of the relationship between patient and RN was important in allowing them to break their unhealthy habits. A good relationship gave patients the confidence to be honest and to talk openly about the problem, an essential step in coming to terms with it. When a lifestyle discussion was experienced as meaningful and as allowing self-determination the discussion provided feelings of support, satisfaction, and confirmation, resulting in a sense of well-being. This is confirmed by previous research showing that without support from the RN, it would have been easy to delay the lifestyle change [6,7,9,17]. If certain demands are placed on patients, they may adopt a passive role, which limits their awareness.

It is easy to assume that close friends and family members play an important role in support, but the patients in our study indicated that they thought it was better to talk with an outsider. A previous study also revealed that overprotective family members could become a source of frustration and make it difficult for the patients to change their lifestyles. Such dynamics have also led to tension in families [5]. The health care system should provide support for those who cannot or do not want to receive support from close relatives. It is important, too, that the health care system provide time and resources for follow-up discussions with the patient, as well as the opportunity for the patient to receive continued support for a period of time after he or she makes a successful change. DeCola et al. [31] indicated that 95% of RNs experience time pressure owing to heavy workloads, and these constraints prevent them from conducting discussions about health promotion with patients. The patients in our study emphasized that long discussions are not necessary, indicating that time can often be found for RNs to talk about lifestyle issues with their patients even during regular visits.

A requirement for health care today is that patients increasingly be allowed to participate in their own care and treatment [32]. Patient autonomy is a key-nursing concept, but it may sometimes be easier for RNs to expect patients to do as they are told [6]. True patient autonomy, however, does not occur until a nurse has fully informed a patient and then supported the patient’s ultimate decision [33]. The patients in our study experienced that the discussions with the RN enabled self-determination, which is an important aspect of patient autonomy. This can open up to a wide use of MI even in discussions that are not about lifestyle. The spirit of MI can, for example, be very useful for health care when the concept of patient autonomy needs to be implemented.

The interviews were used to understand the patients’ experiences of the lifestyle discussions. The follow-up questions asked during the interview process were designed to confirm or clarify the initial answers. Answering open-ended questions and follow-up questions, participants were able to describe their thoughts about the lifestyle discussions in their own words. The study’s validity is enhanced by the fact that all the authors conducted the analysis until agreement on the content and consensus was reached.

In the study we used convenience sampling, which can be seen as a limitation. RNs asked patients who visited them fore lifestyle discussions if they wanted to participate in the study and if the interviewer could call them. This may have resulted in selection bias since the RNs had the opportunity to choose whom they contacted and they may have, intentionally or subconsciously, chosen patients who would favorable report on their experiences. Because the RN initially asked the patient of participation it was also a risk that the patients were dependent on the nurses and may have had difficulty saying no when asked to participate in the study. However, when the interviewer contacted patients to provide more information about the study and about how the interviews would be conducted, each patient was again given the opportunity to withdraw from the study; no one did. Another limitation in the study is that the patients stated that they were motivated and aware of their own role in achieving change. Patients who felt that the discussion style did not suit them or who felt that they were not motivated enough did not continue with the discussions, so our interviews do not include all critical points of view. Failing to sample patients who were exposed to MI but did not return for additional nursing visits i.e. patients with less than three visits, may represent a missed opportunity to learn if there was something about MI that was not appealing to this subgroup.

Patients in this study have been visiting a RN for lifestyle discussions from three to several visits during four years. Those patients who have been on several visits could not specify how many lifestyle discussions they had and the RNs were either not asked how many times each patient had met her for lifestyle discussions, which can be seen as a limitation. Furthermore, there was no feedback from the interviewer to the RN about the interviews with the patients. In order to achieve lifestyle changes Miller & Rollnick [24] describe that patients need to be exposed to a certain “dose” of MI. The number of lifestyle discussions can thus be related to the patients’ experience. Nevertheless the result shows that the patients are unanimous in their positive experience of MI whether they have been on many or few visits, which may be because they have experienced the spirit of MI. This has previously been described by Miller & Rollnick [15] as very important for MI. The result of the present study might therefore be important to better understand patients’ perceptions of provider interactions between the patient and RN when MI is employed.

## Conclusions

The results highlights that nurse-led lifestyle discussions based on MI can enable patients’ self-determination in the process of lifestyle change through the principles of presuming mutual interaction, creating a sense of well-being and contributing to change. This study focuses on MI-based discussions, and the result shows that a subset of patients, who self-reported that they are motivated and aware of their role in making lifestyle changes, appreciate these strategies. However, it is not known whether discussions would be experienced in the same way if RNs used another method or if patients who were less motivated, engaged, or aware of their role in making lifestyle changes were interviewed. Further research comparing various methods for implementing lifestyle changes is needed; such studies should explore the patients’ thoughts in order to reach a deeper understanding of the relevance of specific methods.

## Competing interests

The authors declare that they have no competing interests.

## Authors’ contributions

All authors (EB, SO, HB and CH) contributed to planning of the study. EB undertook the data collection, data analysis and drafting the paper. All authors participated in the data analysis and in revising the paper. All authors read and approved the final manuscript.

## Pre-publication history

The pre-publication history for this paper can be accessed here:

http://www.biomedcentral.com/1472-6955/13/13/prepub
